# Modular Neurodynamics and Its Classification by Synchronization Cores

**DOI:** 10.3389/fnsys.2021.606074

**Published:** 2021-03-12

**Authors:** Frank Pasemann

**Affiliations:** Institute of Cognitive Science, University of Osnabrueck, Osnabrueck, Germany

**Keywords:** connectivity (B), neurodynamics, complexity, synchronization, modularity

## Abstract

It is assumed that the cause of cognitive and behavioral capacities of living systems is to be found in the complex structure-function relationship of their brains; a property that is still difficult to decipher. Based on a neurodynamics approach to embodied cognition this paper introduces a method to guide the development of modular neural systems into the direction of enhanced cognitive abilities. It uses formally the synchronization of subnetworks to split the dynamics of coupled systems into synchronized and asynchronous components. The concept of a synchronization core is introduced to represent a whole family of parameterized neurodynamical systems living in a synchronization manifold. It is used to identify those coupled systems having a rich spectrum of dynamical properties. Special coupling structures—called generative—are identified which allow to make the synchronized dynamics more “complex” than the dynamics of the isolated parts. Furthermore, a criterion for coupling structures is given which, in addition to the synchronized dynamics, allows also for an asynchronous dynamics by destabilizing the synchronization manifold. The large class of synchronization equivalent systems contains networks with very different coupling structures and weights allsharing the same dynamical properties. To demonstrate the method a simple example is discussed in detail.

## 1. Introduction

That the complexity of neural systems, how ever defined, is the source for the cognitive and behavioral capacities of living systems is an almost unquestioned assumption underlying most discussions about the impressive abilities of brains and brain-like systems. Hidden in the term complexity is the question for the still enigmatic structure-function-relationship in these systems. This paper will use a theoretical approach to tackle this problem from the point of modular neurodynamics. It is widely motivated by evolutionary robotics techniques (Nolfi and Floreano, 2000; Harvey et al., 2005; Nolfi et al., 2016), which were applied to study simple neural network solutions for the control of animat behavior (Dean, 1998; Guillot and Meyer, 2001).

If one is using the term complexity in the context of brain-like systems there are at least two different aspects which have to be addressed. One concerns the connectivity of networks; and there are useful measures to quantify what one would term complexity of network connectivity (Sporns et al., 2000; Sporns, 2002). In general, these measures will gain their importance for larger networks.

The other aspect is concerned with the properties of the dynamics running on these neural networks. Complexity in this context will be related to what (Ashby, 1958) called the *law of requisite variety*. Itstates that the larger the variety of actions available to a control system, the larger the variety of perturbations it is able to compensate. This translates into saying that the larger the number of appropriate “states” available to a neural system, the more behavior relevant actions it may generate to cope with occurring vital or life-threatening external situations. To represent these “appropriate states” we identified the so called *dynamical forms* of a neural system (Pasemann, 2017).

A crucial question to ask is that for the relationship between the complexity of the connectivity and the complexity of the dynamics which is realizable on a given network. In the context of brain sciences this has been discussed for instance in Sporns et al. (2000) and Sporns (2002, 2017).

For evolution or development of cognitive neural systems modularity is certainly a good design principle. But already Szentágothai and Arbib (1974), as well as Freeman (1975), formulated the modular architecture of the nervous system as a fundamental organizational principle, where certain brain areas are made up of smaller, repeating neural circuits, which are locally interacting functional units generating the overall performance of the system. Thus, it should be a promising strategy to develop larger neural networks with enriched cognitive faculties by coupling smaller networks in such a way that the resulting system has an extended capacity for sensorimotor control and enhanced cognitive solutions for challenging environmental situations. This is one goal of a modular neurodynamics approach to embodied cognition (Anderson, 2003; Ziemke, 2003).

Concerning “living” systems, neural dynamics is permanently driven by external (and metabolic) sensor signals which can serve, in a first approximation, as (slowly varying) control parameters. For a given set of such parameters the corresponding dynamical system may have a global attractor or a multitude of co-existing attractors forming a so called *attractor landscape*. A small change of parameters may change the whole attractor landscape only marginally: attractors and their basin boundaries are only slightly deformed, they are *morphing* (Negrello and Pasemann, 2008). But at certain critical parameter values the number and/or types of attractors are abruptly changed; i.e., a *bifurcation* occurs. In Pasemann (2017) it was argued, that what is of relevance for behavior is a whole class of parameterized dynamical systems, called a *dynamical form*.

Now, to change behavior (or a mental content) the system should switch from one dynamical form to another dynamical form. In a sensory-motor loop sensor signals can drive parameters across a bifurcation set into a different parameter domain, thus switching to a different dynamical form with a different number and/or different types of attractors. The co-existence of a multitude of attractors in a dynamical form relates to multi-stability, and is functional, for instance, for memory properties. The existence of a larger variety of dynamical forms in a neural structure will enable the system to discern more relevant external (and internal) situations. Moreover, in the spirit of the law of requisite variety, it is a reasonable hypothesis to assume that having more dynamical forms available after the coupling of neuromodules will provide an advantage for the development of cognitive abilities. The goal of coupling subsystems therefore is to enlarge the behavioral or cognitive capacities of neural control systems.

To have a guide line for our analysis we will rely on a set of reasonable assumptions concerning the properties of a network. We will allow neurons to have positive or negative self-connections. Second, the neuromodules which are to be coupled are assumed to be *strongly connected* in the graph theoretical sense, and the coupling of neuromodules is assumed to be *recurrent*, so that the resulting system is again strongly connected. Recurrent coupling will always introduce some additional cycles (in graph theory parlance) into the system and therefore will also influence the dynamics of the composed system.

Synchronization of neuron activities across separated areas of brains was often discussed as a fundamental mechanism underlying cognitive processes. Especially synchronization was understood as a solution to the so called binding problem (Von der Malsburg, 1995; Singer, 1996; Melloni et al., 2007; Maurer, 2016). But its contributions for a live sustaining behavioral performance is still not fully understood and perhaps strongly overestimated (Santos et al., 2012).

Instead of addressing the many roles ascribed to synchronization, in this paper synchronization is considered as a formal tool to understand how synaptic coupling of neuromodules can lead to larger systems with a *richer dynamical spectrum*. The idea here is the following: Suppose that for the composed system already the lower dimensional synchronized dynamics is at least as rich—for instance in terms of the number of attractors—as that of the uncoupled neuromodules. If in addition to this synchronized dynamics there exists a multitude of asynchronous attractors outside the synchronization manifold, then the coupled system is said to have a richer dynamical spectrum than its isolated parts; i.e., the composed system will provide a larger reservoir for different cognitive processes. The goal here, then, is to find criteria for such a situation.

For that we introduce the concept of a *synchronization core* of a composed system. It relates the synchronized dynamics to that of a specific neural network of lower dimension, the dynamics of which may be known. This concept allows to classify a whole family of *synchronization equivalent* neural networks all having different connectivities but carrying qualitatively the same [i.e., topological conjugate (Strogatz, 1996)] synchronization dynamics; i.e., they have the same synchronization core. Concerning the structure-function-relationship in neural systems this approach helps to derive conditions for the dynamical “complexity” of coupled systems.

To enhance the dynamics of given larger neural systems one may think about its decomposition into synchronization equivalent submodules, thus identifying the modules together with their coupling structure. Module connectivities and/or couplings than can be optimized for a more complex neurodynamics.

The organization of the paper is as follows. In the next section a neuromodule is introduced as a parameterized family of discrete-time dynamical systems together with the structure of the underlying neural network. The corresponding concepts of attractor landscapes and dynamical forms are shortly recalled.

Section 3 then will present some basic definitions to characterize the synchronized and asynchronous dynamics of coupled neuromodules (compare also Pasemann, 1999; Pasemann and Wennekers, 2000). It introduces synchronization and obstruction weight matrices, synchronization cores, and discusses the stability of synchronized and asynchronous dynamics and the decomposition of larger networks into synchronizable submodules. The following section 4 then presents a simple example for demonstrating the arguments in the former sections. Finally, the paper concludes with a short discussion of results.

## 2. Neuromodules

Before discussing the role synchronization may play to help understanding the interplay between connectivity and dynamics of coupled neural networks we will introduce some basic notations.

In the following **N**^*n*^(*θ*, *w*) refers to a neural network with *n* neurons, given bias terms *θ* ∈ ℝ^*n*^ and (*n* × *n*) weight matrix *w* ∈ ℝ^*n*×*n*^. The transfer function of neurons is chosen to be the sigmoid *τ* : = tanh.

Although a neuromodule is generally understood as a neural network which can be a part of a larger neural system, here, being interested mainly in the qualitative aspects of dynamics on networks with standard sigmoidal neurons, we will restrict our considerations to discrete-time neurodynamics and describe a *neuromodule* as a parameterized family of dynamical systems A=(A,f;Q), where *A* ⊂ ℝ^*n*^ denotes the *activation state space* of the network, *Q* ⊂ ℝ^(*n*+1)×*n*^ the *parameter space*, and the map *f* : *Q* × *A* → *A* the discrete-time dynamics defined by

(1) $$\begin{array}{l} a_{i}\left(t+1\right)=\theta_{i}+\overset{n}{\underset{j=1}{\sum }}w_{ij}\tau \left(a_{j}\left(t\right)\right),\;i=1,...,n. \end{array}$$

Here, *a*_*i*_ denotes the activation of neuron *i*, *w*_*ij*_ the synaptic weight from neuron *j* to unit *i*, $\theta_{i}={\bar{\theta }}_{i}+I_{i}$ the sum of a fixed bias term ${\bar{\theta }}_{i}$ and its stationary (slowly varying) external input *I*_*i*_. The output *o*_*i*_ of unit *i* is then given by *o*_*i*_ = *τ* (*a*_*i*_) : = tanh(*a*_*i*_); and the *output space*, denoted by A^*^, is corresponds to the open n-cube A^*^ = (−1, 1)^*n*^ ⊂ ℝ^*n*^. Denoting a parameter set by *ρ* : = (*θ*, *w*) ∈ *Q* we also write $A=\left(A,f_{\rho }\right)$ for the dynamics of the underlying neural network **N**^*n*^(*θ*, *w*).

### 2.1. Connectivity, Structure, and Configuration

To better understand the relation between dynamical and network properties we discern between the *connectivity* of a network **N**^*n*^(*θ*, *w*), its *structure*, and its *configuration*.

The *connectivity* of a network **N**^*n*^(*θ*, *w*) is best reflected by its *adjacency matrix* *C*(*w*) which is given by the (*n* × *n*)-matrix with zero elements on the main diagonal and entries *C*_*ij*_(*w*) defined by

(2) $$C_{ij}(w)\;:=\left\{ \begin{array}{l} 1\text{ iff }w_{ij}\neq 0\\ 0\text{ iff }w_{ij}=0\text{ or }i=j\;, \end{array}\right.$$

i.e., the adjacency matrix ignores self-connections (loops) of neurons.

Furthermore, if we want to put emphasis on the fact that there exist inhibitory as well as excitatory synaptic connections in the network **N**^*n*^(*θ*, *w*) we will refer to the *structure matrix* *C*^*S*^(*w*), also called the *structure*, of the network **N**^*n*^(*θ*, *w*) given by

(3) $$C_{ij}^{S}(w)\;:=\left\{ \begin{array}{l} \text{sign}(w_{ij})\text{ iff }w_{ij}\neq 0\\ 0\text{ iff }w_{ij}=0 \end{array}\right.$$

The structure *C*^*S*^(*w*) of a network **N**^*n*^(*θ*, *w*) thus describes the polarity of inter-connections as well as that of self-connections. It is often represented by a signed directed graph. Keeping in mind the underlying neural network **N**^*n*^(*θ*, *w*) of the neuromodule $A=\left(A,f_{\rho }\right)$, we also write $C^{S}\left(A\right)$ for its structure. The structure $C^{S}\left(A\right)$ together with the corresponding family of parameterized dynamical systems $A=\left(A,f_{\rho }\right)$ is what we usually refer to as a *neuromodule*.

An explicitly given weight matrix *w* of a neuromodule $A=\left(A,f_{\rho }\right)$ is called its *configuration*. Finally, the bias terms $\theta =\left(\theta_{1},...,\theta_{n}\right)\in {\mathbb{R}}^{n}$ together with the synaptic weights *w*_*ij*_ of the weight matrix *w* ∈ ℝ^*n*×*n*^ represent the parameters *ρ* ∈ *Q* ⊂ ℝ^(1+*n*)×*n*^ of the neuromodule $A=\left(A,f_{\rho }\right)$.

Thus, given a certain connectivity *C*(*w*) of a network **N**^*n*^(*θ*, *w*), there can be a manifold of different structures *C*^*S*^(*w*) consistent with this connectivity. And there will be a manifold of configurations *w*_*ij*_ for one and the same neural structure *C*^*S*^(*w*).

Given a parameter vector ρ∈Q there may exist not only one attractor, but many attractors of the same type or even of different types. The metaphor *attractor landscape* *L*_ρ_ for ρ∈Q then represents the state space *M* marked by all attractors, their basins of attraction together with their basin boundaries. A *dynamical form* than can be understood as a bundle of such landscapes over a certain set of parameters ρ∈Q for which the corresponding dynamical systems *f*_ρ_ are structurally stable; i.e., topologically conjugate (Pasemann, 2017).

Because a practicable and meaningful complexity measure for our purposes seems to be not yet at hand, we will use the term *richness of the dynamical spectrum* in a purely intuitive way to characterize the dynamical properties of a neuromodule. As an example: a neural network **N**^*n*^(*θ*, *w*) with given weight matrix *w*, allowing only a global fixed point attractor for all bias terms *θ* is assumed to be dynamically less rich than a network for which, for instance, a period doubling route to chaos can be observed in a bifurcation diagram. On the other hand, a network allowing a parameter domain providing a dynamical form with *k* different attractors is termed to be *dynamically richer* than a network providing only *l* < *k* coexisting attractors, where *k* is the maximal number of attractors for all (*A, f*_ρ_), *ρ* ∈ *Q* inherited by a given structure *C*^*S*^(*w*).

### 2.2. Coupled Neuromodules

In the following an n-dimensional neuromodule A=(A,f;Q) is assumed to be given by a *strongly connected* neural network **N**^*n*^(*θ*, *w*); i.e., its weight matrix *w* is assumed to be irreducible.

Let A=(A,f;Q) and $B=\left(B,g;Q^{\prime }\right)$ denote two neuromodules with (*n* × *n*)-weight matrices *w*^*A*^ and *w*^*B*^, respectively. Their corresponding parameter sets are denoted by *ρ*^*A*^ = (*θ*^*A*^, *w*^*A*^) ∈ *Q* and *ρ*^*B*^ = (*θ*^*B*^, *w*^*B*^) ∈ *Q*′, and the neural activations of module A and B will be denoted by *a*_*i*_ and *b*_*i*_, *i* = 1, ..., *n*, respectively.

A system composed of A and B corresponds to a neuromodule M=(M,h;R), with *M* : = (*A* × *B*), *h* : *R* × *M* → *M*, and (*Q* × *Q*′) ⊂ *R*; i.e., the new parameters space *R* includes the parameters of Q and $Q^{\prime }$ and is enlarged by the weights of synaptic connections between neurons of the two modules A and B.

The connections from module B to module A are described by an (*n* × *n*)-*coupling matrix* *w*^*AB*^, connections from A to B by (*n* × *n*)-*coupling matrix* *w*^*BA*^. Thus, the (2*n* × 2*n*) weight matrix *w*^*M*^ of the coupled system M is of the form

(4) $$\begin{array}{l} w^{M}\;:=\left(\begin{array}{cc} w^{A} & w^{AB}\\ w^{BA} & w^{B} \end{array}\right). \end{array}$$

The pair (*w*^*AB*^, *w*^*BA*^) of matrices comprising the *coupling connections* is simply called the *coupling* of modules A and B. It is called *recurrent* if *w*^*AB*^ ≠ 0 and *w*^*BA*^ ≠ 0. A recurrent coupling (*w*^*AB*^, *w*^*BA*^) is called *symmetric*, iff *w*^*AB*^ = *w*^*BA*^. Recurrent couplings will lead again to strongly connected neural networks M with irreducible weight matrix *w*^*M*^; i.e., to neuromodules.

## 3. Dynamics of Coupled Neuromodules

The dynamics of coupled neuromodules will depend on the strength of the coupling connections and on their type, i.e., being excitatory or inhibitory. It will also depend on the type (even or odd) and length of the cycles established by the coupling connections. Recall, that a cycle is termed even (odd) if the number of inhibitory connections in the cycle is even (odd). To get some first results concerning the effect of different couplings we assume that the neuromodules A and B have the same dimension *n*. For the more general case where modules have different dimensions see e.g., Pasemann and Wennekers (2000).

In the following M=(M,h;R) denotes a system of coupled *n*-modules A and B with weight matrix *w*^*M*^ given by Equation (4). The activation state space *M* = *A* × *B* of the coupled system is 2*n*-dimensional, and its parameterized discrete-time dynamics will be denoted by

$$h_{\rho }:M\to M,\;\rho \in R,$$

where *ρ* : = (*ρ*^*A*^, *ρ*^*B*^, *w*^*A*^, *w*^*B*^, *w*^*AB*^, *w*^*BA*^) denotes a set of parameters for the coupled system (*M, h*_*ρ*_). The dynamics *h*_*ρ*_ is then given for *i* = 1, . . . , *n* in the form

(5) $$\begin{array}{l} a_{i}\left(t+1\right)=\theta_{i}^{A}+\overset{n}{\underset{j=1}{\sum }}w_{ij}^{A}\tau \left(a_{j}\left(t\right)\right)+\overset{n}{\underset{j=1}{\sum }}w_{ij}^{AB}\tau \left(b_{j}\left(t\right)\right), \end{array}$$

(6) $$\begin{array}{l} b_{i}\left(t+1\right)=\theta_{i}^{B}+\overset{n}{\underset{j=1}{\sum }}w_{ij}^{B}\tau \left(b_{j}\left(t\right)\right)+\overset{n}{\underset{j=1}{\sum }}w_{ij}^{BA}\tau \left(a_{j}\left(t\right)\right). \end{array}$$

**Definition** Given a coupled system M=(M,h;R). Suppose there exist a subset D⊂M, such that $\left(a_{0},b_{0}\right)\in D$ implies

(7) $$\begin{array}{l} \underset{t\to \infty }{\lim}|\left(a_{i}\left(t;a_{0}\right)\right)-\kappa \cdot b_{i}\left(t;b_{0}\right)|=0,\;i=1,...,n,\;\kappa \in \left[-1,1\right], \end{array}$$

where (*a*(*t*; *a*_0_), *b*(*t*; *b*_0_)) denotes the orbit in *M* under *h*_*ρ*_ through the initial condition $\left(a_{0},b_{0}\right)\in D$. Then this process is called a (complete) *synchronization* of modules A and B if κ = 1. If κ = −1 the dynamics is called (completely) *anti-synchronous*. Otherwise the dynamics is called *asynchronous*.

### 3.1. Synchronized Module Dynamics

Let M=(M,h;R) denote the system of coupled modules A=(A,f;Q) and $B=\left(B,g;Q^{\prime }\right)$ which can be completely synchronized. Then the synchronized dynamics is restricted to the *synchronization manifold* *M*^*s*^ which is an *n*-dimensional hyperspace *M*^*s*^ ≅ ℝ^*n*^ ⊂ *M*. One can immediately verify the following lemma by subtracting Equation (6) from Equation (5); i.e., by equating *a*_*i*_ = *b*_*i*_, *i* = 1, . . . , *n*.

**Lemma 3.1**. *Let the parameter sets* *ρ*^*A*^ ∈ *Q*, *ρ*^*B*^ ∈ *Q*′ *of the modules* A *and* B *satisfy*

(8) $$\begin{array}{l} \theta^{A}=\theta^{B},\;\left(w^{A}-w^{BA}\right)=\left(w^{B}-w^{AB}\right),\;w^{BA}\neq 0,\;w^{AB}\neq 0. \end{array}$$

*Then every orbit of* *h*_*ρ*_ : *M* → *M* *with initial condition* *a*(0) = *b*(0) ∈ *M*^*s*^ *is constrained to* *M*^*s*^ *for all times; i.e.,* *M*^*s*^ *is an invariant manifold for* *h*_*ρ*_.

Equation (8) is called the *synchronization condition* for coupled neuromodules, and it shows that synchronization can be achieved for modules with different weight matrices *w*^*A*^ and *w*^*B*^, as well as with different coupling matrices *w*^*AB*^ and *w*^*BA*^, as long as (8) is satisfied.

For a more detailed study of the synchronized dynamics it is appropriate to introduce new coordinates (ξ, η) for *M* which are parallel and orthogonal, respectively, to the synchronization manifold *M*^*s*^:

(9) $$\begin{array}{l} \xi_{i}\;:=\frac{1}{2}\left(a_{i}+b_{i}\right),\;\eta_{i}\;:=\frac{1}{2}\left(a_{i}-b_{i}\right),\;i=1,...,n. \end{array}$$

An orbit on the synchronization manifold *M*^*s*^ then will be given by *η*_*i*_ = 0, *i* = 1, . . . , *n*. Furthermore, the hyperspace *M*^⊥^ ⊂ *M* defined by *ξ*_*i*_ = 0, *i* = 1, . . . , *n*, will be called the *obstruction manifold*.

In terms of the (*ξ*, *η*)-coordinates the general dynamics *h*_*ρ*_ : *M* → *M* of the coupled system then reads

(10) $$\begin{array}{l} \xi_{i}(t+1)=\frac{1}{2}\;\cdot \;(\theta_{i}^{A}+\theta_{i}^{B})+\frac{1}{2}\sum_{j=1}^{n}(w_{ij}^{A}+w_{ij}^{BA})\;\cdot \;G^{+}(\xi_{j}(t),\eta_{j}(t))\\ \;+\frac{1}{2}\sum_{j=1}^{n}\left(w_{ij}^{B}+w_{ij}^{AB}\right)\;\cdot \;G^{-}(\xi_{j}(t),\eta_{j}(t))\;, \end{array}$$

(11) $$\begin{array}{l} \eta_{i}(t+1)=\frac{1}{2}\;\cdot \;(\theta_{i}^{A}-\theta_{i}^{B})+\frac{1}{2}\;\sum_{j=1}^{n}\left(w_{ij}^{A}-w_{ij}^{BA}\right)\;\cdot \;G^{+}(\xi_{j}(t),\eta_{j}(t))\\ \;-\frac{1}{2}\;\sum_{j=1}^{n}\left(w_{ij}^{B}-w_{ij}^{AB}\right)\;\cdot \;G^{-}(\xi_{j}(t),\eta_{j}(t)))\;, \end{array}$$

where *i* = 1, . . . , *n*, and the functions *G*^±^ are defined by

(12) $$\begin{array}{l} G^{\pm }\left(x,y\right)\;:=\tau \left(x\pm y\right),\;x,y\in \mathbb{R}. \end{array}$$

These functions have the following properties:

(13) $$\begin{array}{l} G^{\pm }\left(x,0\right)=\tau \left(x\right), \end{array}$$

(14) $$\begin{array}{l} G^{+}\left(0,y\right)=-G^{-}\left(0,y\right)=\tau \left(y\right), \end{array}$$

(15) $$\begin{array}{l} \partial_{x}G^{+}\left(x,y\right){|}_{y=0}=\partial_{x}G^{-}\left(x,y\right){|}_{y=0}=\tau^{\prime }\left(x\right), \end{array}$$

(16) $$\begin{array}{l} \partial_{y}G^{+}\left(x,y\right){|}_{y=0}=-\partial_{y}G^{-}\left(x,y\right){|}_{y=0}=\tau^{\prime }\left(x\right), \end{array}$$

where *τ′* denotes the derivative of the sigmoid *τ* = tanh. Recall that 0 < *τ′*(*x*) ≤ 1, *x* ∈ ℝ, and *τ′*(0) = 1 so that the linearization *Dh*_*ρ*_ of the general dynamics *h*_*ρ*_ at the origin 0 ∈ *M* is equal to the weight matrix *w*^*M*^ of the coupled system; i.e.,

$$Dh_{\rho }\left(0\right)=w^{M}.$$

Defining the *synchronization matrix* *w*^+^ by

(17) $$\begin{array}{l} w^{+}\;:=\left(w^{A}+w^{AB}\right)=\left(w^{B}+w^{BA}\right), \end{array}$$

and setting

$$\theta \;:=\theta^{A}=\theta^{B},$$

the *synchronized dynamics* $h_{\rho }^{s}:M^{s}\to M^{s}$ is given, in terms of the *ξ*-coordinates, by the *n* equations

(18) $$\begin{array}{l} \xi_{i}\left(t+1\right)=\theta_{i}+\overset{n}{\underset{j=1}{\sum }}w_{ij}^{+}\cdot \tau \left(\xi_{j}\left(t\right)\right),\;i=1,...,n. \end{array}$$

Thus, the synchronized dynamics $h_{\rho }^{s}$ corresponds to that of an *n*-module with weight matrix *w*^+^ depending on the bias terms *θ* : = *θ*^*A*^ = *θ*^*B*^. It may have fixed point attractors as well as periodic, quasiperiodic, or chaotic attractors, all constrained to *M*^*s*^. Although the persistence of the synchronized dynamics is guaranteed by the synchronization conditions (8), it is not at all clear that the synchronized dynamics (18) is *asymptotically stable*, in the sense, that small perturbations will not desynchronize the system. Thus, an orbit in *M* may be an attractor for the synchronized dynamics $f_{\rho }{|}_{M^{s}}$ but not for the global dynamics *f*_*ρ*_ of the coupled system (Ashwin et al., 1996).

Furthermore, if the synchronization conditions (8) are satisfied then the corresponding *obstruction dynamics* $h_{\rho }^{⊥}:M^{⊥}\to M^{⊥}$ is given in terms of the η-coordinates by the equations

(19) $$\begin{array}{l} \eta_{i}\left(t+1\right)=\overset{n}{\underset{j=1}{\sum }}w_{ij}^{-}\cdot \tau \left(\eta_{j}\left(t\right)\right),\;i=1,...,n, \end{array}$$

where *w*^−^ denotes the *obstruction matrix* defined by

(20) $$\begin{array}{l} w_{ij}^{-}\;:=\left(w_{ij}^{A}-w_{ij}^{BA}\right)=\left(w_{ij}^{B}-w_{ij}^{AB}\right). \end{array}$$

Observe that the obstruction dynamics $h_{\rho }^{⊥}$ does not depend on bias terms, as long as *θ*^*A*^ = *θ*^*B*^. This means that the origin *η*^0^ ∈ *M*^⊥^ is always a fixed point for the *η*-dynamics (19). As long as *η*^0^ ∈ *M*^⊥^ is an asymptotically stable fixed point for $h_{\rho }^{⊥}$ the corresponding synchronized dynamics will be asymptotically stable. Recall that the obstruction matrix *w*^−^ is identical with the linearization of the obstruction dynamics at the origin; i.e., $Dh_{\rho }^{⊥}\left(\eta^{0}\right)=w^{-}$.

Usually asymptotic stability of *M*^*s*^ is discussed in terms of the *synchronization Liapunov exponents* $\lambda_{i}^{s}$ and the *transversal Liapunov exponents* $\lambda_{i}^{⊥}$, *i* = 1, . . . , *n*, for the synchronized dynamics $h_{\rho }^{s}$ on *M*^*s*^, as for instance in Pasemann and Wennekers (2000). There it was shown that the synchronization manifold *M*^*s*^ is asymptotically stable, if the largest transversal Lyapunov exponent $\lambda_{1}^{⊥}$ satisfies $\lambda_{1}^{⊥}<0$ for all orbits in *M*^*s*^. But since this is the case if all eigenvalues *ϵ*_*i*_ of the obstruction matrix *w*^−^ satisfy |*ϵ*_*i*_| < 1, *i* = 1, . . . , *n*, the asymptotic stability of the synchronization manifold *M*^*s*^ can be controlled by the obstruction matrix *w*^−^ alone.

Thus, to reserve the full dynamical spectrum provided by an *n*-module with weight matrix *w*^+^ for a pure synchronized dynamics of a coupled system one just has to choose a coupling (*w*^*AB*^, *w*^*BA*^) such that the moduli of the eigenvalues of the corresponding obstruction matrix *w*^−^ are all smaller than 1 (compare the example in section 4, **Figure 12**).

To get a reasonable estimate for the weights ${w_{ij}}^{-}$ one can apply for instance a theorem like the one in Hammer and Tiňo (2003) to obtain

** Theorem 3.2**. *Given an n-dimensional obstruction dynamics* $\left(M^{⊥},h_{\rho }^{⊥}\right)$ *with respect to an obstruction matrix* *w*^−^. *If*

$$\underset{ij}{\max}\left(w_{ij}^{-}\right)<\frac{1}{n},\;i,j=1,...,n,$$

*then the origin* *η*^0^ ∈ *M*^⊥^ *is a global fixed point attractor for* $h_{\rho }^{⊥}$, *and the corresponding synchronization dynamics* $h_{\rho }^{s}$ *is asymptotically stable*.

Following the Liapunov exponent approach as in Pasemann and Wennekers (2000) one will trivially derive

** Lemma 3.3**. *Let* **N**^*n*^(*θ*, *w*) *be a synchronizable neuromodule with synchronization matrix* *w*^+^ *and obstruction matrix* *w*^−^. *Let* *ϵ*_*i*_, *i* = 1, . . . , *n*, *denote the eigenvalues of the obstruction matrix* *w*^−^. *If*

(21) $$\begin{array}{l} \underset{i}{\max}|\epsilon_{i}|<1, \end{array}$$

*then the synchronization manifold* *M*^*s*^ *is asymptotically stable*.

Correspondingly, to destabilize a synchronization manifold *M*^*s*^, one has to chose a coupling (*w*^*AB*^, *w*^*BA*^) such that the eigenvalues of the obstruction matrix *w*^−^ are non-zero with moduli large enough to make contributions to the positivity of the largest transversal exponent $\lambda_{1}^{⊥}$.

### 3.2. Synchronization Cores

Being interested again not only in the synchronized dynamics of a specific configuration of coupled neuromodules, which depends on a given set of parameters (*θ*, *w*) ∈ *R*, but in the full dynamical spectrum of synchronization available for coupled neuromodules we refer to the following

**Definition** Given a system M of coupled modules A and B with coupling (*w*^*AB*^, *w*^*BA*^) satisfying (8). The structure *C*^*S*^(*w*^+^) of its synchronization matrix *w*^+^ is called the *synchronization core* of the coupled system M.

There can be many different structures and module configurations of A and B with many different coupling matrices *w*^*AB*^ and *w*^*BA*^ all leading to the same synchronization core. An example can be seen in section 4 **Figure 14** where three different structures all have the same synchronization core *w*^+^ ∈ ℝ^*n* × *n*^ shown in **Figure 5** (right). All these different modules and different couplings will lead to the same parameterized family of synchronized dynamics. Therefore, at the same time a synchronization core *w*^+^ stands for a whole class of synchronizable neural networks of dimension *n*, and we define

**Definition** Two coupled systems M and $M^{\prime }$ with synchronization cores *C*^*S*^(*w*^+^) and *C*^*S*^(*w*^′+^) are called *synchronization equivalent* if they have the same synchronization core; i.e., there exists a coupling (*w*^*AB*^, *w*^*BA*^) such that

$$C^{S}\left(w^{+}\right)=C^{S}\left(w\prime^{+}\right).$$

With respect to the structure-function relationship in coupled systems one then can ask for the effects different couplings (*w*^*AB*^, *w*^*BA*^) will have for the resulting dynamics on the modular neural network. To answer this question the following definition is introduced.

**Definition** Given a system M of coupled modules A and B with coupling matrices *w*^*AB*^ and *w*^*BA*^ satisfying (8). Let *C*^*S*^(*w*^+^) denote its synchronization core. If there exists at least one pair of indices (*i, j*) such that $w_{ij}^{+}\neq 0$ but $w_{ij}^{A}=0$ and $w_{ij}^{B}=0$, then the corresponding coupling (*w*^*AB*^, *w*^*BA*^) is called *generative*. Otherwise it is called *conservative*.

A synchronization core *C*^*S*^(*w*^+^) may be identical to the structures *C*^*S*^(*w*^*A*^) or *C*^*S*^(*w*^*B*^) of the isolated modules A or B, or it may be of different type. If it is generative it represents a family of parameterized dynamical systems different from those of the modules A and B. And it may allow for a richer dynamical spectrum than that of the isolated parts A and B.

**Figure 1:**
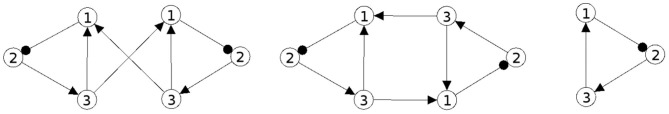
Conservative coupling of two odd 3-cycles (left), the same structure but twisted (middle), and the synchronization core *C*^*S*^(*w*^+^) (right).

**Figure 2:**
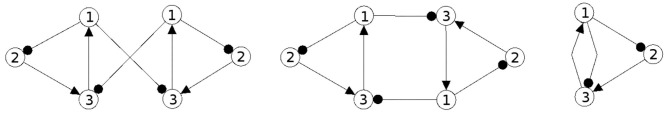
Generative coupling of two odd 3-cycles (left), the same structure but twisted (middle), and the synchronization core *C*^*S*^(*w*^+^) (right).

In Figures 1, 2 examples of a conservative and of a generative coupling of two odd 3-cycles are shown (left). For odd 3-cycles one observes, besides global fixed point attractors, only one dynamical form with co-existing period-2 and period-6 attractors (Pasemann, 1995). To illustrate the differences, the graphs are twisted (middle) revealing that a generative coupling introduces an additional 4-cycle into the system. That this coupling is generative can be seen by the synchronization core (right).

Although in the first case the synchronization core is again an odd 3-cycle, in the generative case one observes a new odd 2-cycle in the synchronization core. The resulting 3-dimensional synchronized dynamics in the 6-dimensional network now displays many new periodic attractors up to quasi-periodicity, as simulation experiments show. This supports the hypothesis that generative couplings of neuromodules can enable a richer dynamical spectrum than that of the isolated modules.

### 3.3. Decomposition of Neural Networks

Having used synchronization so far as a tool to compose larger neural systems from smaller neuromodules to derive a richer dynamical spectrum one may now ask if it can also be used to determine how larger systems can be configured in such a way that their dynamical spectrum is enhanced. For that we try to decompose a large network into two sub-modules which can be completely synchronized. One can then optimize the system by choosing an appropriate coupling (*w*^*AB*^, *w*^*BA*^) which gives the desired properties of the corresponding synchronization and obstruction matrices *w*^+^ and *w*^−^. So first we have

**Definition** Given a neuromodule **N**^*n*^(*θ*, *w*) of even dimension *n* = 2*m* with structure *C*^*S*^(*w*). It is called decomposable into synchronizable submodules, or *s-decomposable*, if there exist two recurrent sub-modules A and B with structures *C*^*S*^(*w*^*A*^) and *C*^*S*^(*w*^*B*^), and a coupling (*w*^*AB*^, *w*^*BA*^) which satisfies the synchronization condition (8) such that

(22) $$\begin{array}{l} w=\left(\begin{array}{cc} w^{A} & w^{AB}\\ w^{BA} & w^{B} \end{array}\right). \end{array}$$

Equation (22) is called an *s-decomposition* of **N**^*n*^(*θ*, *w*). A neuromodule **N**^*n*^(*θ*, *w*), *n* = 2*m*, is called *basic* if it is not s-decomposable.

**Definition** Given an s-decomposable neuromodule **N**^*n*^(*θ*, *w*) with submodules A and B, coupling (*w*^*AB*^, *w*^*BA*^), and synchronization matrix *w*^+^. Its s-decomposition is called *generative* if the synchronization matrix *w*^+^ is generative. Otherwise it is called *conservative*. A neural network is called *basic* if it is not s-decomposable.

Under the assumed condition of complete synchronization, it is clear that an s-decomposable neuromodule **N**^*n*^(*θ*, *w*) must be of even dimension. But s-decompositions may be generalized also to partial synchronized neuromodules (Pasemann and Wennekers, 2000).

**Figure 3:**
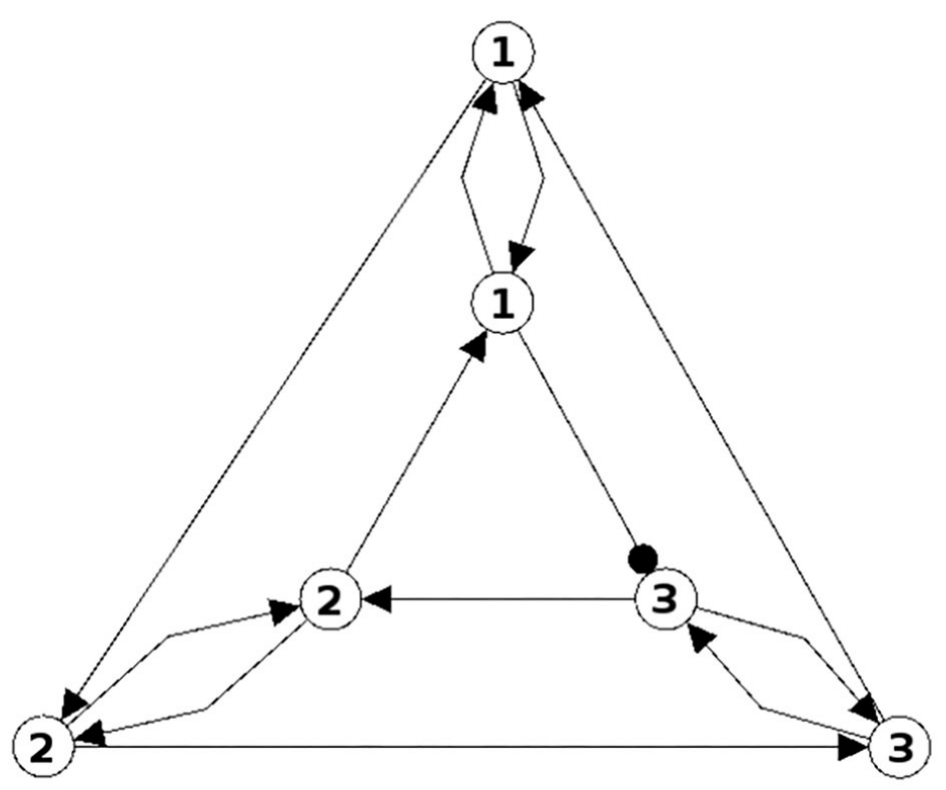
A six-neuron module which is *not* s-decomposable into two three-neuron modules. It is a basic module.

**Figure 4:**
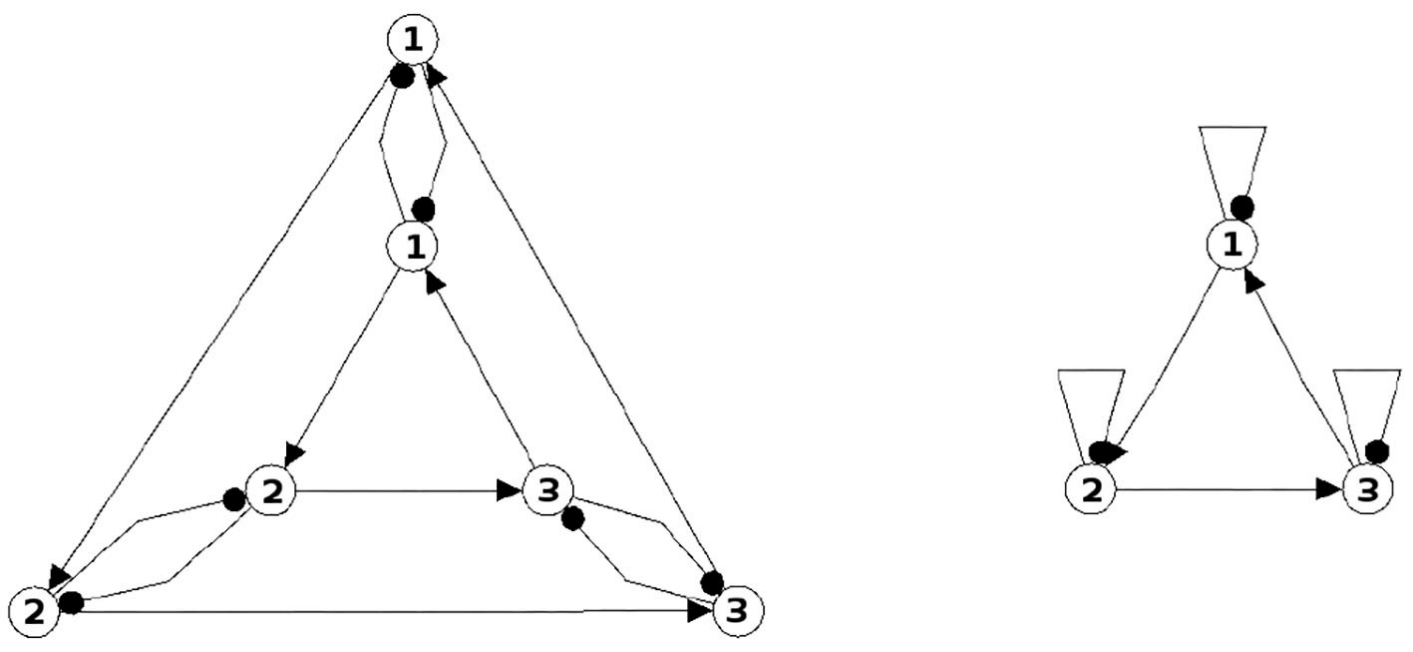
A six-neuron neuromodule (left) which is s-decomposable and the resulting synchronization core *C*^*S*^(*w*^+^) (right), showing that its s-decomposition is *generative*.

The following figures show examples of a basic module with six neurons (Figure 3), and an s-decomposable module with its synchronization core *C*^*S*^(*w*^+^), demonstrating that this s-decomposition is generative (Figure 4).

Analyzing the generative synchronization core *C*^*S*^(*w*^+^) of the s-decom-posable module in Figure 4, one observes again that the synchronized dynamics has a richer dynamical spectrum than the isolated 3-modules. For instance, the core *C*^*S*^(*w*^+^) allows for quasiperiodic attractors, although the parts, that is the basic (even or odd) 3-cycles can display only r-periodic attractors with *r* = 1, 3 and *r* = 2, 6, respectively.

The identification of neural structures that are s-decomposable or basic may help to develop networks with a desired rich dynamical spectrum. Proving conjectures like the following can help to find larger classes of such structures:

- If the connectivity of a neural network corresponds to the Cayley graph of a finitely generated Abelian group, then it is s-decomposable.
- If the connectivity of a neural network does not contain a cycle of even length, then it is not s-decomposable.

## 4. A Simple Example

**Figure 5:**
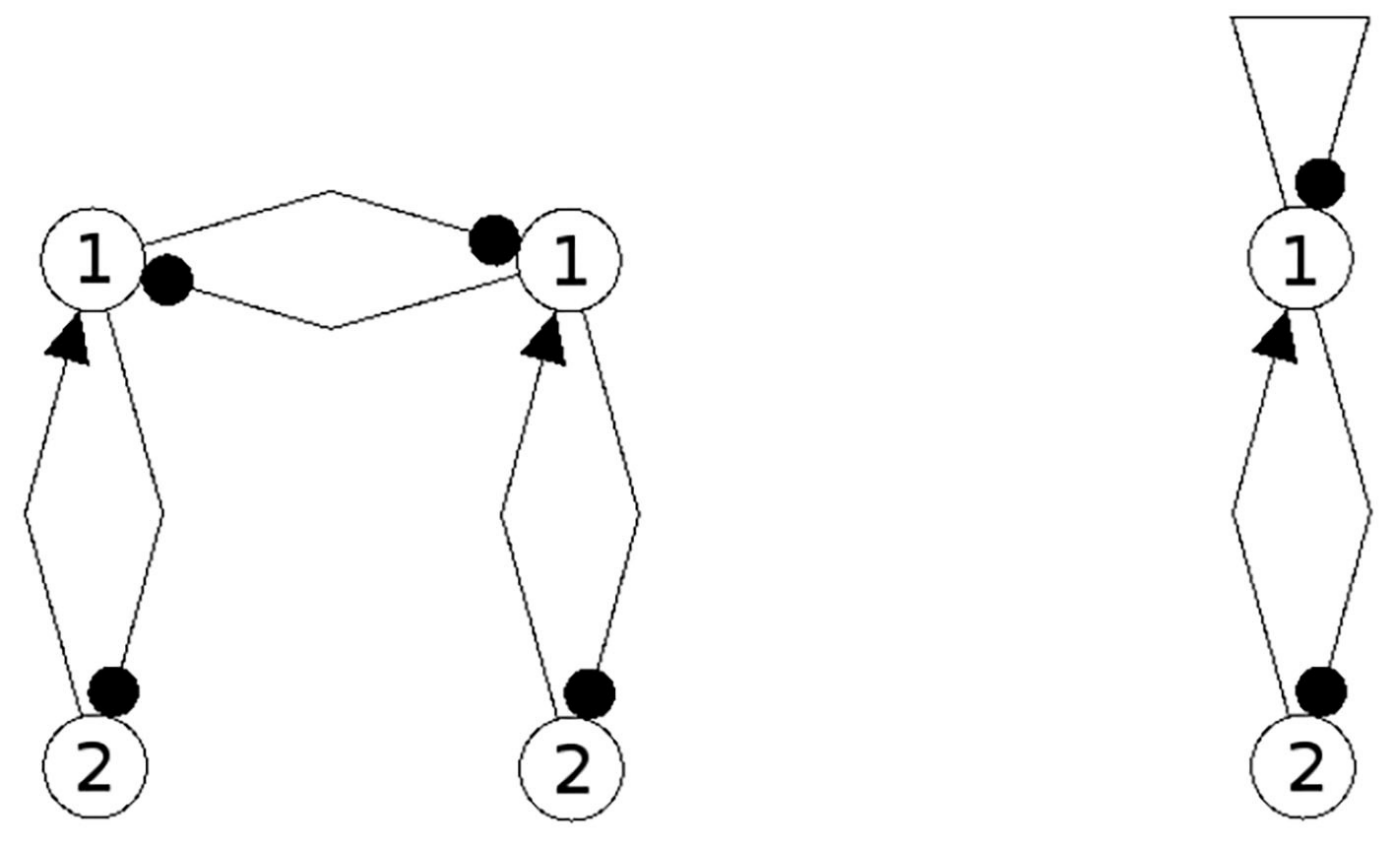
The structure $C^{S}\left(M\right)$ of the coupled system M (left) and its synchronization core *C*^*S*^(*w*^+^) (right).

Some interesting features of coupled neural networks can be studied already for the special case of coupling *identical* modules. For our discussion this is not an essential restriction, but allows a simpler notation. To illustrate the foregoing theoretical arguments we therefore choose the following simple example. Coupled are two identical odd 2-cycles without self-couplings (compare Figure 5) leading to a 4-dimensional neural network M=(M,h;R). With respect to the synchronization condition (8 the notation then reduces to bias terms satisfying

(23) $$\begin{array}{l} \theta_{1}\;:=\theta_{1}^{A}=\theta_{1}^{B},\;\theta_{2}\;:=\theta_{2}^{A}=\theta_{2}^{B}, \end{array}$$

and with *w* := *w*^*A*^ = *w*^*B*^ one has

(24) $$\begin{array}{l} w_{11}=w_{22}=0,\;w_{12}=-w_{21}\neq 0. \end{array}$$

Such odd 2-cycles come in only two dynamical forms: one with a global fixed point attractor, and one with a global period-4 attractor.

The recurrent coupling *w*^*coup*^ = *w*^*BA*^ = *w*^*AB*^ is chosen to have only one non-zero element

$$w_{11}^{coup}=\;w_{11}^{AB}=\;w_{11}^{BA}<0,$$

so that the synchronization and obstruction matrices read

(25) $$\begin{array}{l} w^{+}=\left(\begin{array}{cc} w_{11}^{coup} & w_{12}\\ w_{21} & 0 \end{array}\right),\;w^{-}=\left(\begin{array}{cc} -w_{11}^{coup} & w_{12}\\ w_{21} & 0 \end{array}\right). \end{array}$$

This leads to the structure $C^{S}\left(M\right)$ of the composed system M depicted in Figure 5 (left) and to its synchronization core *C*^*S*^(*w*^+^) (right). One observes that the recurrent coupling *w*^*coup*^ is generative: it generates a negative self-connection of neuron 1 which was absent in the original 2-neuron networks. But structures like the synchronization core *C*^*S*^(*w*^+^) allow for configurations which display periodic, quasi-periodic and even chaotic attractors (Pasemann, 2002), so that already the synchronized dynamics of the coupled system M has a much richer dynamical spectrum than the original odd 2-cycles.

To go into deeper analysis we consider first the stability properties of the synchronized dynamics $h_{\rho }^{s}$ on *M*^*s*^. Because here the eigenvalues of *w*^+^ and *w*^−^ have identical moduli, from section 3.1 we know that the largest synchronization exponent here is equal to the largest transversal exponent:

(26) $$\begin{array}{l} \lambda_{1}^{s}=\lambda_{1}^{⊥}. \end{array}$$

Thus, for $\lambda_{1}^{⊥}>0$ orbits will never be asymptotically stable on all of *M*; and in fact, synchronized chaos will always be hyperchaotic.

To analyze this situation we choose the weights of the synchronization matrix *w*^+^ large enough to allow for chaotic dynamics by setting

(27) $$\begin{array}{l} w^{+}=\left(\begin{array}{cc} -4 & 1.6\\ -1.6 & 0 \end{array}\right), \end{array}$$

which is obtained by setting

(28) $$\begin{array}{l} w=\left(\begin{array}{cc} 0 & 1.6\\ -1.6 & 0 \end{array}\right),\;w^{coup}=\left(\begin{array}{cc} -4 & 0\\ 0 & 0 \end{array}\right). \end{array}$$

The obstruction matrix then reads

(29) $$\begin{array}{l} w^{-}=\left(\begin{array}{cc} 4 & 1.6\\ -1.6 & 0 \end{array}\right). \end{array}$$

**Figure 6:**
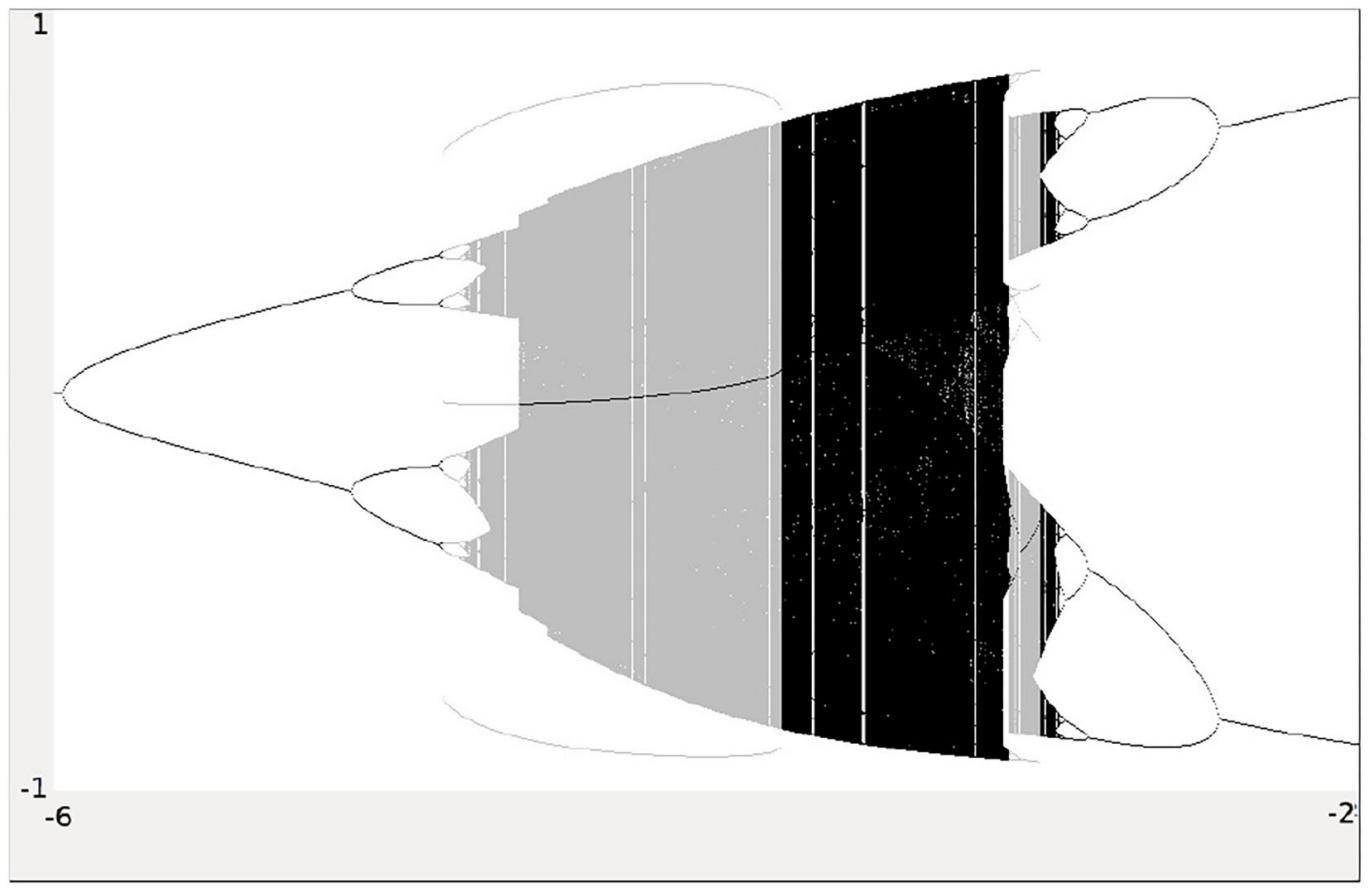
Bifurcation diagram for the varied bias term *θ*_1_ of the synchronous dynamics according to *w*^+^ given by (27). The bias term *θ*_2_ = 0 is fixed. Shown is the mean output $\bar{o}=0.5\cdot \left(o_{1}+o_{2}\right)$ of the 2-neuron module corresponding to the synchronization core *C*^*S*^(*w*^+^) in Figure 5 (right). Coexisting attractors are marked by gray shaded domains.

Analyzing the synchronous dynamics using the synchronization matrix (27) and fixing *θ*_2_ = 0 one realizes in the corresponding bifurcation diagram for *θ*_1_ (Figure 6) a chaotic domain around −4.75 < *θ*_1_ < −3.1.

**Figure 7:**
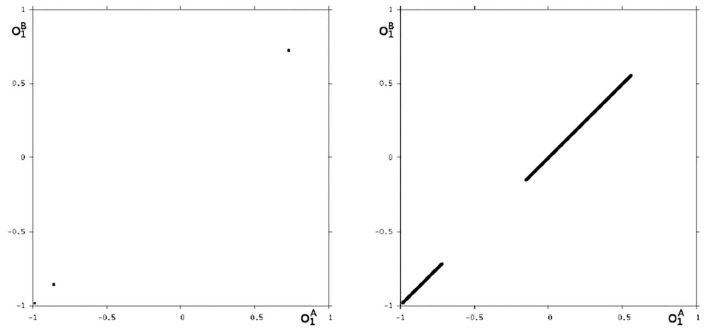
The two synchronized attractors in *M*^*s*^ for *θ*_1_ = −4: A period-3 and a chaotic attractor. Shown are their projections to the $\left(o_{1}^{A},o_{1}^{B}\right)$-output space.

**Figure 8:**
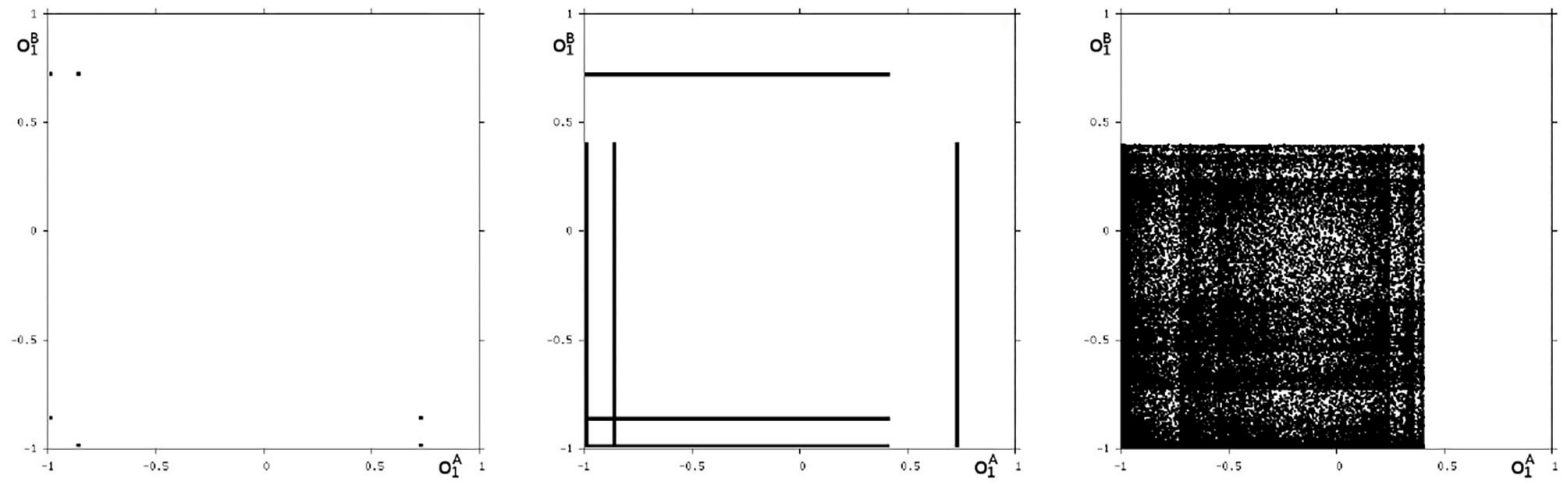
The three asynchronous attractors in *M* for *θ*_1_ = −4: a period-6, and two chaotic attractors. Shown are their projections to the $\left(o_{1}^{A},o_{1}^{B}\right)$-output space.

Because this configuration has quite interesting dynamical properties and may serve as an example for what was called a “rich dynamical spectrum,” we will identify the coexistent attractors for certain bias values *θ*_1_ from this interval. For that we use their projections onto the $\left(o_{1}^{A},o_{1}^{B}\right)$-output space. As suggested by the bifurcation diagram in Figure 6 we check the situation for *θ*_1_ = −4 and find five different attractors for this configuration: a period-3 and a chaotic attractor in the synchronization manifold *M*^*s*^ shown in Figure 7, and an asynchronous period-6 attractor together with two asynchronous chaotic attractors, as can be seen in Figure 8. They are derived by using random initial conditions in the 4-dimensional output space M^*^. One further observes that all asynchronous attractors are symmetric with respect to the synchronization manifold *M*^*s*^, which is typical for coupled identical modules (Pasemann, 1999).

**Figure 9:**
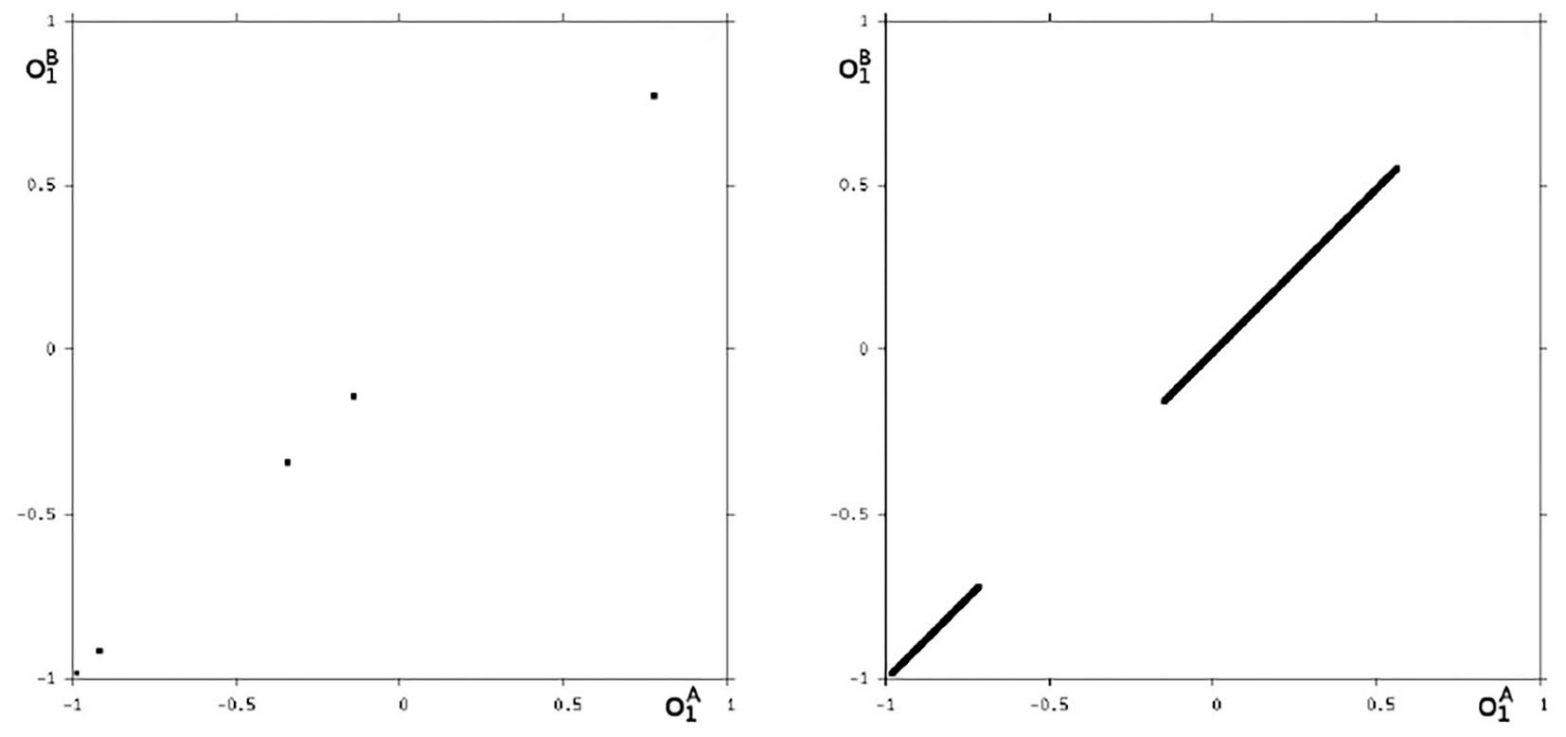
The two synchronized attractors for *θ*_1_ = −3: a period-5 and a chaotic attractor. Shown are their projections to the $\left(o_{1}^{A},o_{1}^{B}\right)$-output space.

**Figure 10:**
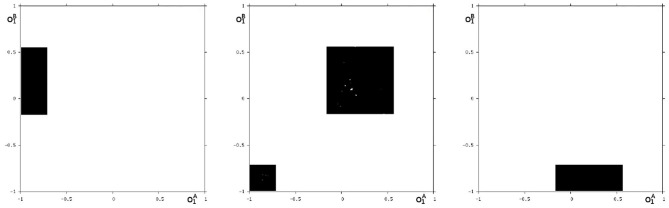
Three asynchronous chaotic attractors for *θ*_1_ = −3. Shown are their projections to the $\left(o_{1}^{A},o_{1}^{B}\right)$-output space.

**Figure 11:**
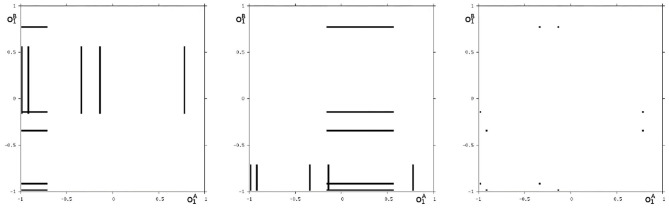
The additional two asynchronous chaotic attractors for *θ*_1_ = −3, and an asynchronous period-10 attractor for *θ*_1_ = −3. Shown are their projections to the $\left(o_{1}^{A},o_{1}^{B}\right)$-output space.

We may also have a look at the situation for *θ*_1_ = −3.0 where coexisting attractors can be assumed according to the shaded structure in Figure 6. Using again randomly chosen initial conditions on m^*^ we are able to identify at least eight different coexisting attractors: In the synchronization manifold *M*^*s*^ there co-exists a period-5 attractor together with a chaotic attractor (Figure 9). Furthermore there are six asynchronous attractors, one of them is a period-10 attractor, the other five attractors are chaotic. The projections to the $\left(o_{1}^{A},o_{1}^{B}\right)$-output space of all these attractors are displayed in Figures 10, 11.

### 4.1. Globally Stable Synchronization Manifolds

If one wants to keep the full dynamical spectrum of the synchronization core *C*^*S*^(*w*^+^) with *w*^+^ as in Equation (27), but wants to keep the dynamics being strictly synchronous for all varying *θ*, one has to choose a configuration such that the synchronization manifold *M*^*s*^ is globally stable. This is guarantied if the obstruction matrix *w*^−^, according to section 3.1, has eigenvalues all satisfying |λ_*i*_| < 1, *i* = 1, . . . , *n*. For that, instead of (29), one may choose an obstruction matrix

(30) $$\begin{array}{l} w^{-}=\left(\begin{array}{cc} -1.35 & 0.6\\ -0.6 & 0 \end{array}\right), \end{array}$$

which has eigenvalues −0.984 and −0.366, respectively. Then one may define a convenient weight matrix *w* = *w*^*A*^ = *w*^*B*^ for the modules and calculate the corresponding coupling *w*^*coup*^ = *w*^*AB*^ = *w*^*BA*^ as in the following

(31) $$\begin{array}{l} w=\left(\begin{array}{cc} -2.675 & 1.1\\ -1.1 & 0 \end{array}\right),\;w^{coup}=\left(\begin{array}{cc} -1.325 & 0.5\\ -0.5 & 0 \end{array}\right). \end{array}$$

**Figure 12:**
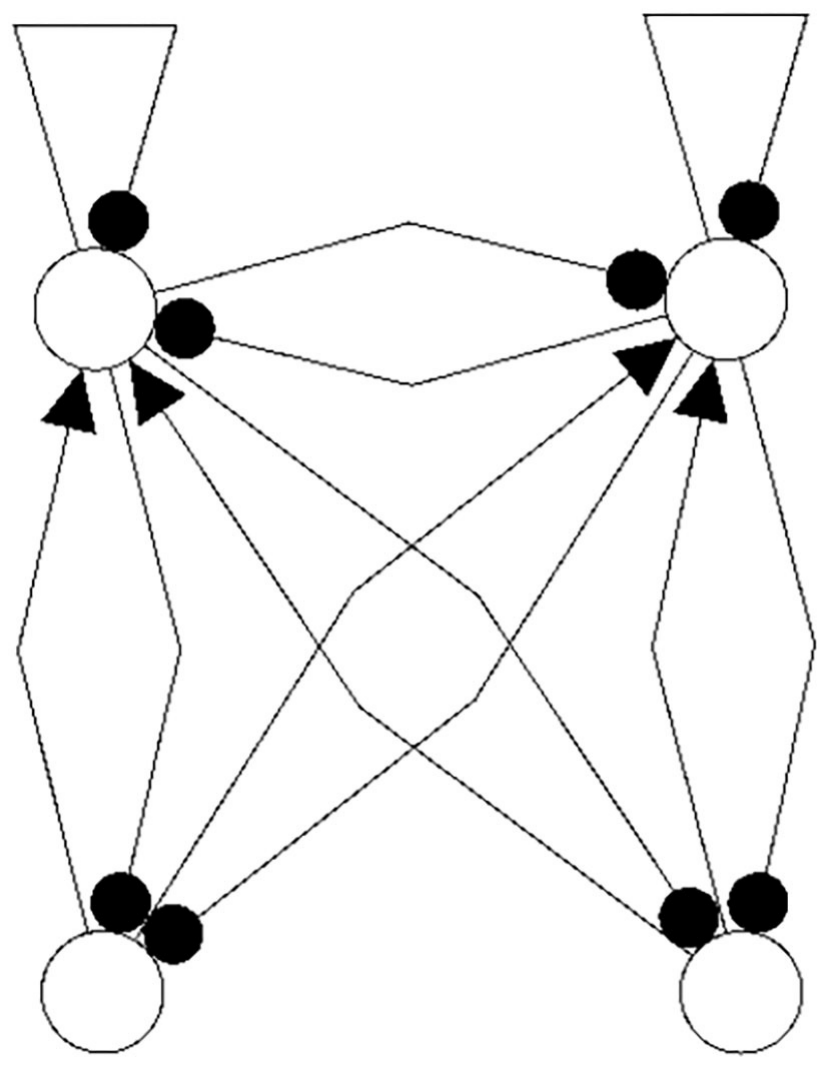
The example of a conservative coupling of 2n-modules allowing the same dynamical spectrum corresponding to the synchronization matrix *w*^+^ (27) in a globally stable synchronization manifold *M*^*s*^.

The corresponding configuration is shown in Figure 12 having the same synchronization core *C*^*S*^(*w*^+^) as the structure discussed above (Figure 5).

### 4.2. Discussion of the Example; Generative Couplings

Although the example of this section is very simple, the dynamics for two specific parameter values, characterized in the figures above, clearly demonstrate what we termed a “rich dynamical spectrum.” And it also makes clear that this richness is the direct cause of the generative coupling of the two odd 2-cycles. Furthermore, the strong coupling ${w_{11}}^{coup}=-4$ leads to a large modulus of the eigenvalues of the synchronization matrix *w*^+^ and also of the obstruction matrix *w*^−^ given in (29). Therefore, also the largest transversal Lyapunov exponent satisfies $\lambda_{1}^{⊥}>0$ and therefore the synchronization manifold *M*^*s*^ is destabilized; but, recall, orbits with initial conditions in the synchronization manifold *M*^*s*^ will stay in *M*^*s*^. With initial conditions outside of *M*^*s*^ one observes additional asynchronous attractors in the state space *M* of the coupled system, even if the synchronization condition (8) is satisfied. Thus, it depends essentially on the initial conditions whether the coupled system M will follow a synchronous or asynchronous orbit.

Further analysis shows that smaller negative coupling values $w_{11}^{coup}$ will drive the synchronized dynamics of the coupled system M into a domain with quasi-periodic attractors. But because the modulus of the largest eigenvalue of *w*^−^ remains still large enough to destabilize the synchronization manifold *M*^*s*^ there will still exist also some asynchronous attractors in *M*. In fact, besides a synchronized quasi-periodic attractor in *M*^*s*^ there is a multitude of asynchronous quasi-periodic attractors in *M* for certain values of the bias term *θ*_1_. Even if one changes the strength of the connections *w*_12_ = −*w*_21_ the synchronization manifold *M*^*s*^ will stay unstable, and there exist some asynchronous attractors as well until the modulus of the largest eigenvalue of *w*^−^ is smaller than 1.0. This demonstrates that a generative coupling guaranties over a large *θ*-parameter domain co-existing synchronous as well as asynchronous attractors.

**Figure 13:**
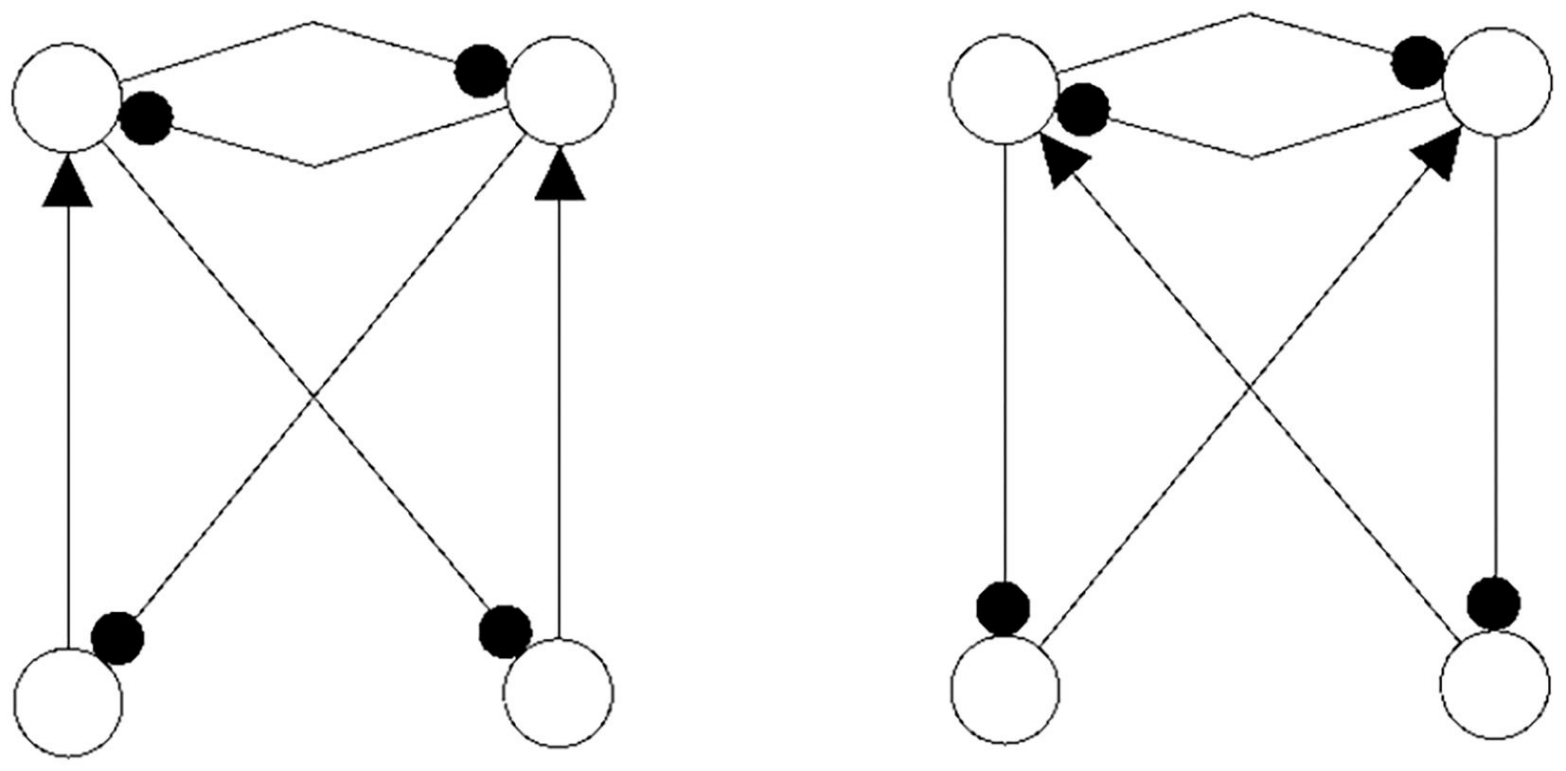
Coupled feedforwardnetworks resulting in the same synchronization core of this example.

It should be mentioned here that one may even couple non-recurrent networks, like the feedforward networks in Figure 13, to obtain the same synchronization core *C*^*S*^(*w*^+^) as in our example, Figure 5 right. So synchronization cores are well defined not only for coupled neuromodules but, more generally, also for coupled non-recurrent networks. To observe the same synchronized dynamics of such systems it is only necessary that they have the same synchronization matrix *w*^+^ and obstruction matrix *w*^−^. This indicates that in general the class of synchronization equivalent networks comprises a large number of structures that are all able to carry exactly the same types of synchronized dynamics. And that generative couplings of “simple” networks can lead to networks with a much richer dynamical spectrum than that observed for the original subsystems.

### 4.3. Discussion of the Example; Conservative Couplings

**Figure 14:**
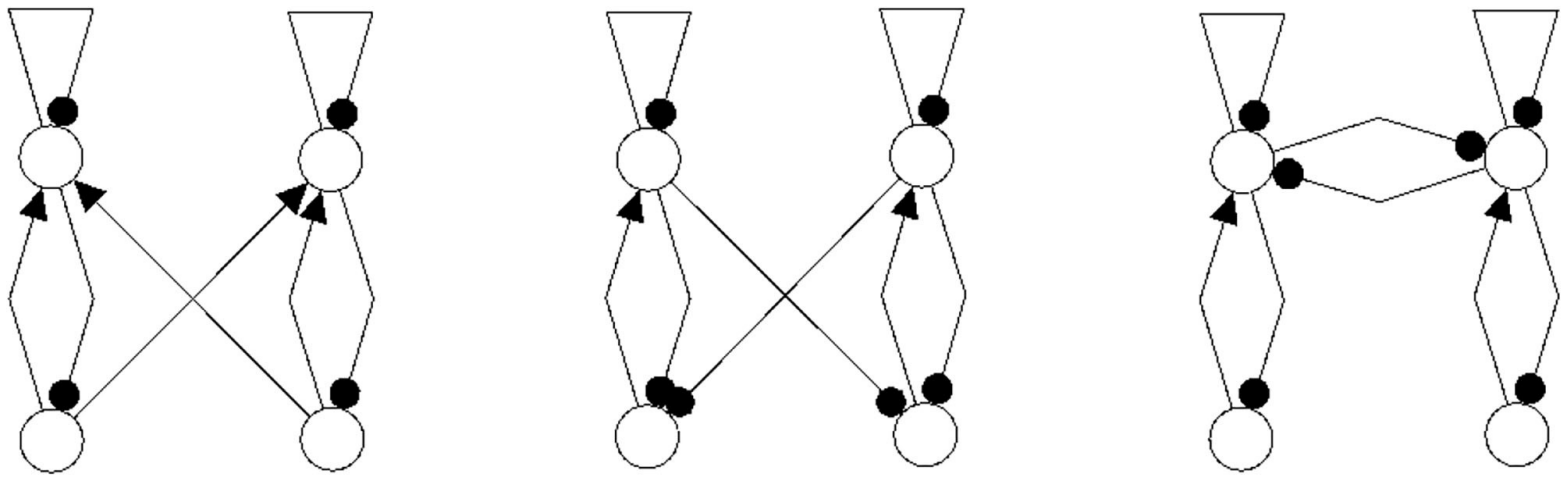
Examples of network structures all having the same synchronization core *C*^*S*^(*w*^+^) as our example shown in Figure 5 right. They all have the same parameterized family of 2-dimensional synchronized dynamics.

Now then, what is the effect of conservative couplings? Because the result of these couplings is a synchronization core *C*^*S*^(*w*^+^), which is the same as the structure of the parts $C^{S}\left(A\right)=C^{S}\left(B\right)$, it will not generate a synchronized dynamics with a richer dynamical spectrum than that of the parts. Examples of such non-generative couplings, all having the same synchronization core *C*^*S*^(*w*^+^) as our example (Figure 5), are shown in Figure 14. One therefore can infer that all the corresponding different network configurations will have a synchronized dynamics which is identical with the synchronized dynamics discussed in the example above. But here the dynamical spectrum is identical with that of the coupled 2-neuron networks (Pasemann, 2002).

### 4.4. Discussion of the Example; Relation to K-Sets

**Figure 15:**
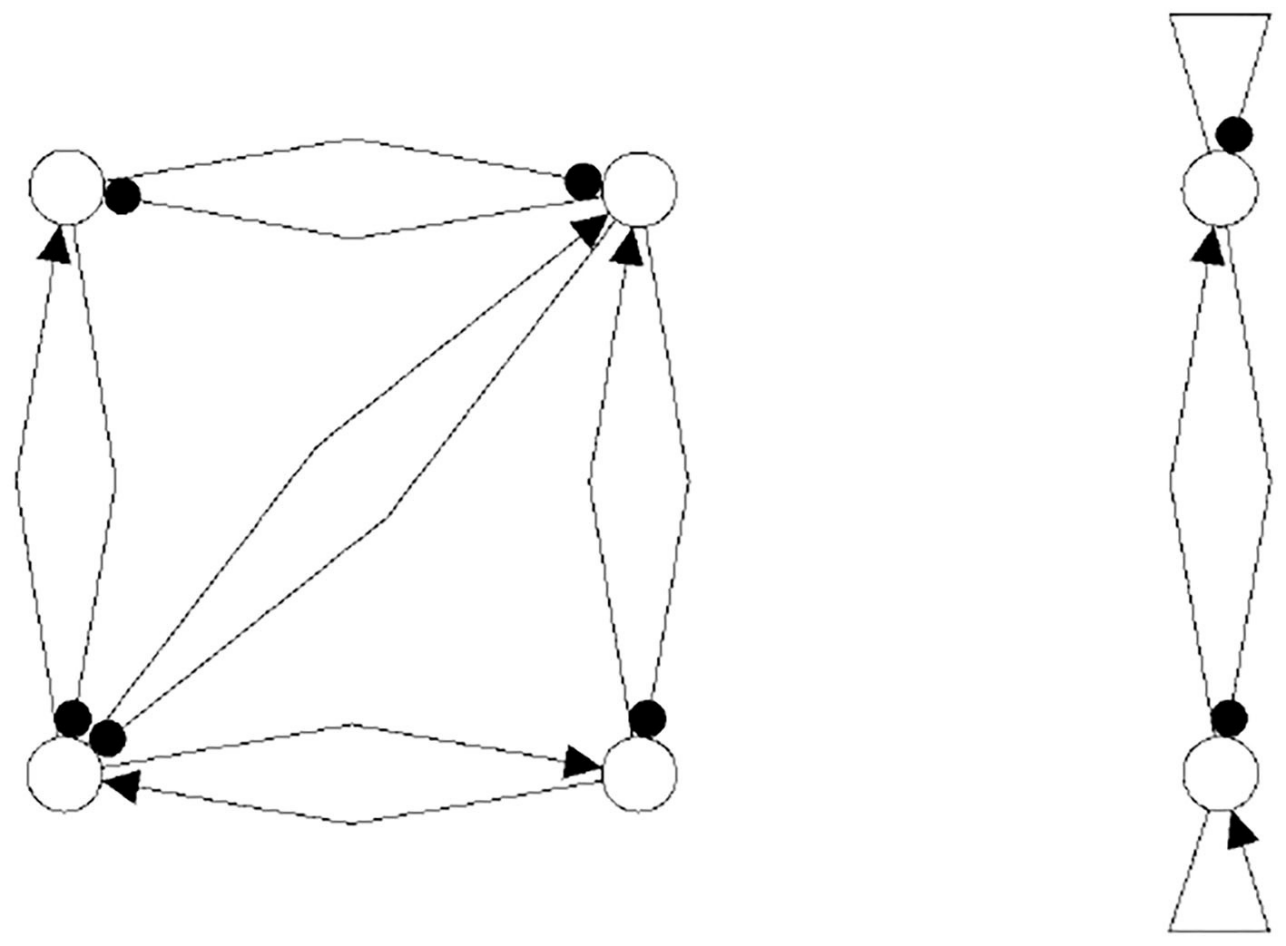
The structure of a Freeman KII-set *C*^*S*^(KII) (left) and its synchronization core *C*^*S*^(*w*^′+^) (right).

One may further remark that, based on the neurophysiological findings, Freeman (1975) identified 10 basic neurodynamical modules, the Katchalsky K-sets, that help to explain how neural populations can create the complex dynamics essential for cognition. One of the simplest modules, called the Freeman KII-set, consists of two excitatory and two inhibitory populations. The structure *C*^*S*^(KII) of a KII-set is displayed in Figure 15 (left) together with its synchronization core *C*^*S*^(*w*^′+^) (right). Although this synchronization core has an additional positive self-connection, the synchronized dynamics of the KII-set is not qualitatively different from that of the example discussed above, i.e., it has the same dynamical spectrum. Having this in mind, it is not a surprise that a corresponding structure for artificial neurons served as a versatile module for dynamic memory designs, for robust classification, for pattern recognition, and for navigation tasks (Kozma, 2008; Kozma and Freeman, 2009).

## 5. Summary

Based on the assumption that cognitive abilities of brains and brain-like systems rest on their dynamical properties, the development of artificial neural networks providing cognitive abilities calls for systems having a manifold of different non-trivial attractors between which can be switched by external (and internal) sensor signals (Pasemann, 2017).

As a guideline for generating neural systems with such a desired “rich dynamical spectrum,” for instance by applying an evolutionary algorithm, we propose to couple synchronizable subsystems with a certain dynamical spectrum to obtain a neural network with an even richer dynamical spectrum. For two neuromodules of dimension *n*, represented by their signed connections (structures), which can be synchronized by applying appropriate interconnections, we observe that the resulting synchronized dynamics of the 2*n*-dimensional network is identical to that of an *n*-dimensional neuromodule, defined by the synchronization matrix *w*^+^ and named the *synchronization core* *C*^*S*^(*w*^+^).

Using so called *generative couplings*, even the *n*-dimensional synchronized dynamics may have a richer dynamical spectrum than the isolated *n*-dimensional subsystems. Furthermore, based on the *obstruction matrix* *w*^−^ criteria are given for the co-existence of asynchronous attractors outside of the synchronization manifold, so that at the same time there exist attractors for the synchronized dynamics as well as for an asynchronous dynamics; i.e., the dynamical spectrum of the composed system is much richer. Thus, a network composed according to these rules will provide a larger variety of attractors for use as memories or for action control. For applications, as usual, which attractor goes into actions depends on bias terms (slow sensor inputs), the crossing of bifurcation manifolds or applied initial conditions (Pasemann, 2017).

To demonstrate the basic ideas, we concentrated on the recurrent coupling of two identical 2-dimensional neuromodules, two 2-cycles. Although this is an extremely simple configuration we made visible a manifold of co-existing synchronous and asynchronous attractors for what was a generative coupling. For such 2 × 2-dimensional networks also conservative (non-generative) couplings where discussed, and an example provides the presumpsion that the concept of generative couplings can be extended to not strongly recurrent, but still synchronizable subsystems. Also a comparison with Freeman KII-sets, which were derived from neurophysiological findings, is given.

From our results one can infer that the class of synchronization equivalent neural structures contains very many even dimensional networks, all having the same properties of their synchronous dynamics. And, furthermore, networks displaying a rich dynamical spectrum do not need to have many connections. This observation may be related to the small world view of network functionality (Watts and Strogatz, 1998).

Although concepts of the paper relate only to completely synchronized even dimensional composed systems, a generalization to partially synchronized neural networks (Pasemann and Wennekers, 2000) is straight forward.

The ultimate goal of this approach is to develop strategies for selecting reasonable neuromodules which can serve as individuals for the initial population of an evolutionary neuro-robotics run. Evolutionary programs, like for instance the Interactively Constrained Neuro-Evolution (ICONE) (Rempis and Pasemann, 2012) program, are prepared to use populations of neuromodules (instead of neurons) and coupling connections as basic elements for an evolution of behavior relevant neural control-networks for autonomous robots.

## Data Availability Statement

The raw data supporting the conclusions of this article will be made available by the authors, without undue reservation.

## Author Contributions

The author confirms being the sole contributor of this work and has approved it for publication.

## Conflict of Interest

The author declares that the research was conducted in the absence of any commercial or financial relationships that could be construed as a potential conflict of interest.
